# Establishing Methods to Monitor Influenza (A)H5N1 Virus in Dairy Cattle Milk, Massachusetts, USA

**DOI:** 10.3201/eid3113.250087

**Published:** 2025-05

**Authors:** Elyse Stachler, Andreas Gnirke, Kyle McMahon, Michael Gomez, Liam Stenson, Charelisse Guevara-Reyes, Hannah Knoll, Toni Hill, Sellers Hill, Katelyn S. Messer, Jon Arizti-Sanz, Fatinah Albeez, Elizabeth Curtis, Pedram Samani, Natalia Wewior, David H. O’Connor, William Vuyk, Sophia E. Khoury, Matthew K. Schnizlein, Nicole C. Rockey, Zachariah Broemmel, Michael Mina, Lawrence C. Madoff, Shirlee Wohl, Lorraine O’Connor, Catherine M. Brown, Al Ozonoff, Daniel J. Park, Bronwyn L. MacInnis, Pardis C. Sabeti

**Affiliations:** Broad Institute of MIT and Harvard, Cambridge, Massachusetts, USA (E. Stachler, A. Gnirke, K. McMahon, M. Gomez, L. Stenson, C. Guevara-Reyes, H. Knoll, T. Hill, S. Hill, K.S. Messer, J. Arizti-Sanz, F. Albeez, E. Curtis, P. Samani, N. Wewior, A. Ozonoff, D.J. Park, B.L. MacInnis, P.C. Sabeti); University of Puerto Rico-Rio Piedras, San Juan, Puerto Rico, USA (C. Guevara-Reyes); Harvard University, Cambridge (P. Samani, B.L. MacInnis, P.C. Sabeti); University College London, London, UK (P. Samani); University of Wisconsin-Madison, Madison, Wisconsin, USA (D.H. O’Connor, W. Vuyk); The University of Texas at Austin, Austin, Texas, USA (S.E. Khoury); Michigan State University, East Lansing, Michigan, USA (M.K. Schnizlein); Duke University, Durham, North Carolina, USA (N.C. Rockey, Z. Broemmel); Immune Observatory, Boston, Massachusetts, USA (M. Mina); University of Massachusetts Chan Medical School, Worcester, Massachusetts, USA (L.C. Madoff); Massachusetts Department of Public Health, Boston (L.C. Madoff, S. Wohl, C.M. Brown); Brigham and Women’s Hospital, Boston (S. Wohl); Massachusetts Department of Agricultural Resources, Boston (L. O’Connor); Boston Children’s Hospital, Boston (A. Ozonoff); Harvard Medical School, Boston (A. Ozonoff); Howard Hughes Medical Institute, Chevy Chase, Maryland, USA (P.C. Sabeti)

**Keywords:** influenza, viruses, cattle, milk, influenza A virus, H5N1 subtype, viral RNA, farms, workflow, PCR, disease outbreak, public health, public health surveillance, United States

## Abstract

Highly pathogenic avian influenza A(H5N1) virus has caused a multistate outbreak among US dairy cattle, spreading across 16 states and infecting hundreds of herds since its onset. We rapidly developed and optimized PCR-based detection assays and sequencing protocols to support H5N1 molecular surveillance. Using 214 retail milk samples from 20 states for methods development, we found that H5N1 virus concentrations by digital PCR strongly correlated with quantitative PCR cycle threshold values; digital PCR exhibited greater sensitivity. Metagenomic sequencing after hybrid selection was best for higher concentration samples, whereas amplicon sequencing performed best for lower concentrations. By establishing these methods, we were able to support the creation of a statewide surveillance program to perform monthly testing of bulk milk samples from all dairy cattle farms in Massachusetts, USA, which remain negative to date. The methods, workflow, and recommendations described provide a framework for others aiming to conduct H5N1 surveillance efforts.

Highly pathogenic avian influenza A(H5N1) virus infection has caused large-scale outbreaks in wild and domestic birds, resulting in mass deaths, culling events, and economic losses (*1*). Viral spillover to mammals has become more frequent, including outbreaks involving mammal-to-mammal transmission and sporadic human infections (*2*). In March 2024, H5N1 clade 2.3.4.4b virus was found in unpasteurized milk produced by infected dairy cattle in the United States, the first confirmation of an outbreak that grew to span 927 herds in 16 states as of January 15, 2025 (*3*,*4*). The outbreak subsequently spread through interstate transport of cattle, milking practices, and shared milking machinery and farm equipment (*5*,*6*). Although confirmed human cases have thus far been sporadic and have primarily been associated with mild symptoms, the spread of H5N1 virus in cattle threatens the dairy industry and risks further adaptation to mammalian hosts, including humans.

This outbreak has highlighted the need for rapidly deployable H5N1 molecular surveillance capacity to detect infections, monitor viral spread and evolution, identify transmission routes, and target interventions to protect agricultural assets and food supply and prevent broader human transmission. Cow milk has emerged as an ideal sample source for H5N1 virus detection and surveillance during this outbreak; the virus is shed in high concentrations in milk, likely because of its affinity for infecting mammary gland epithelial cells (*7*). However, milk undergoes intense processing steps, including ultrapasteurization and homogenization, which have unknown effects on viral RNA quality.

We optimized methods for nucleic acid extraction, molecular detection, and sequencing of H5N1 virus in cow milk, first using synthetic nucleic acid material and subsequently validating those methods by using positive retail milk samples from affected states. By quickly establishing a robust workflow for detecting and sequencing H5N1 virus from milk as the outbreak emerged, we were positioned to support mandatory statewide surveillance for H5N1 virus in milk from dairy cattle farms across Massachusetts. This program, launched in August 2024, was implemented preemptively in the absence of H5N1 detection in the state and surrounding region to confirm the absence of H5N1 and to serve as an early warning system if a local outbreak occurs. State authorities worked with farms to collect samples from bulk milk tanks from all 95 dairy cattle farms across Massachusetts, initially within a 3-week period, followed by a rotating sampling schedule testing all farms monthly. On the basis of our workflow development and validation using retail milk samples (see next section), we extracted bulk milk samples using the MagMAX CORE extraction kit (Thermo Fisher Scientific, https://www.thermofisher.com) and performed digital PCR (dPCR) to detect H5N1 virus; we used the bovine RNaseP gene (RP_Bov) as a positive internal control. Although the surveillance program is ongoing, we have completed 4 rounds of statewide testing, and H5N1 has not been detected in the state. The RP_Bov–positive control has been routinely detected at similar levels to retail milk, providing confidence in the negative results obtained for H5N1 (Appendix Figure 1).

The sensitivity of our workflow allows for preemptive surveillance of H5N1 for the typical size of a Massachusetts dairy cattle farm (≈10,000 cows on 125 farms) (*8*). On the basis of our limit of detection (LOD) of 10^4^ copies/mL milk, to detect 1 infected cow in a herd size of either 100 or 1,000 cows, the infected cow would have to be shedding 10^6^ H5N1 copies/mL milk (for a herd of 100) or 10^7^ H5N1 copies/mL milk (for a herd of 1,000). This level is within the concentration range of live virus shed by infected cattle (10^4^–10^8.8^ 50% tissue culture infectious dose/mL) (*7*). Despite the complexity of milk as a sample type, the robust detection of viral RNA in affected milk offers a unique surveillance mechanism to easily monitor lactating herds by testing pooled bulk milk tank samples, saving time and resources compared with the testing of individual cows.

## Characteristics of the Validated Workflow

This article is meant to serve as a resource documenting how other laboratories can quickly validate and implement testing. The characteristics of the validated workflow are summarized next (Appendix). First, we tested performance of a previously published H5N1 assay targeting the H5 subtype of the hemagglutinin (HA) gene inclusive of the current virus outbreak strain (*9*) (H5_Taq) by both quantitative PCR (qPCR) and dPCR (Appendix). We optimized primer and probe concentrations using synthetic H5N1 RNA, selecting for optimal linearity, sensitivity, accuracy, precision, and qPCR efficiency (Appendix Figures 2, 3).

**Figure 1:**
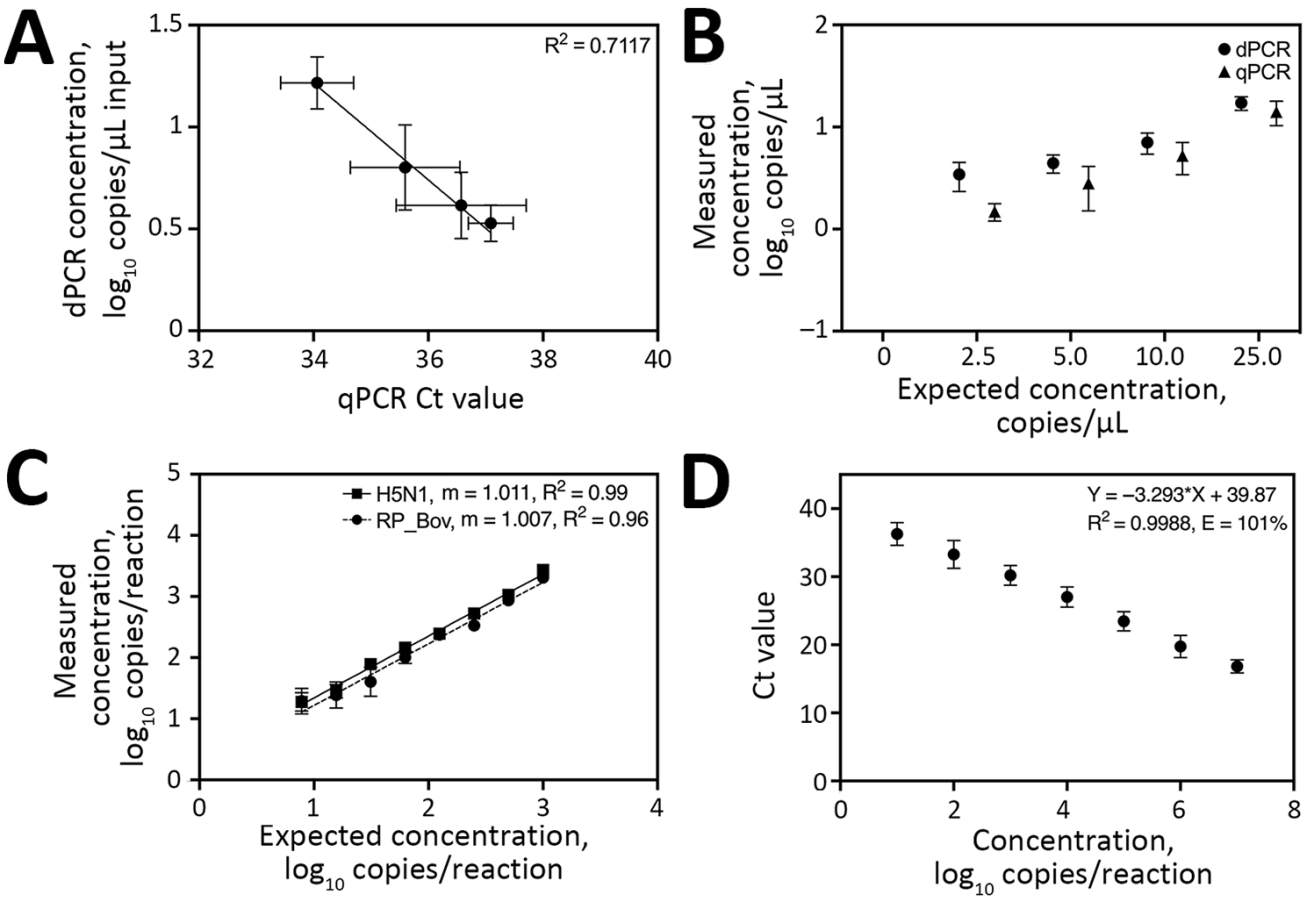
Validation and characterization of dPCR and qPCR on synthetic spike-in samples in study of methods to monitor influenza A(H5N1) virus in dairy cattle milk, Massachusetts, USA. A, B) Limit of detection analysis for correlation of dPCR concentrations with qPCR Ct values (A) and measured concentrations compared to expected concentrations for both qPCR and dPCR (B). C, D) Detection of dPCR (H5_Taq and RP_Bov) (C) and qPCR (H5_Taq) (D) assays using serial dilutions of synthetic H5N1 RNA standard material. For qPCR data, we combined and jointly analyzed all standard curve data from runs during retail milk testing. Fitted lines in panels A and D represent simple linear regression lines of best fit. Error bars indicate +1 SD. Ct, cycle threshold; dPCR, digital PCR; qPCR, quantitative PCR; R^2^, coefficient of determination.

Overall, the H5N1 assay displayed robust performance on both platforms; dPCR outperformed qPCR in LOD and precision. The 90% LOD was 5 copies/μL by dPCR and 10 copies/μL by qPCR. In addition, although dPCR concentrations correlated well with qPCR cycle threshold (Ct) values (Figure 1, panel A), dPCR exhibited lower coefficients of variations, ranging from 10.5% to 26.4%, compared with 18.0% to 111.5% for qPCR (Figure 1, panel B). Both assays maintained linearity over their dynamic ranges (Figure 1, panels C, D).

As a positive internal control for nucleic acid extraction in cattle milk, we designed a PCR targeting the bovine Ribonuclease P gene (both DNA and RNA; RP_Bov). By dPCR, linearity was maintained across all dilutions tested (Figure 1, panel C) with a 90% LOD of 10 copies/μL. On the basis of the superior performance of dPCR for the H5N1 target virus, the RP_Bov assay was not evaluated as a qPCR. Overall, all PCRs performed well with minimal optimization.

We next evaluated preprocessing and extraction protocols to optimize sample preparation for subsequent H5N1 virus detection and sequencing. We tested 2 commercially available extraction kits, MagMAX Prime Viral/Pathogen (Prime) and MagMAX CORE (CORE) (both Thermo Fisher Scientific), by spiking serial dilutions of synthetic H5N1 nucleic acid into milk. We tested milk with various fat contents and examined the effect of pre-centrifugation (at either 1,200 × *g* or 12,000 × *g*) on outcomes. We also tested the MagMAX Wastewater kit (Wastewater) (Thermo Fisher Scientific) head-to-head with the CORE kit on a subset of 8 retail milk samples previously found to be H5N1 virus positive with CORE kit testing.

**Figure 2:**
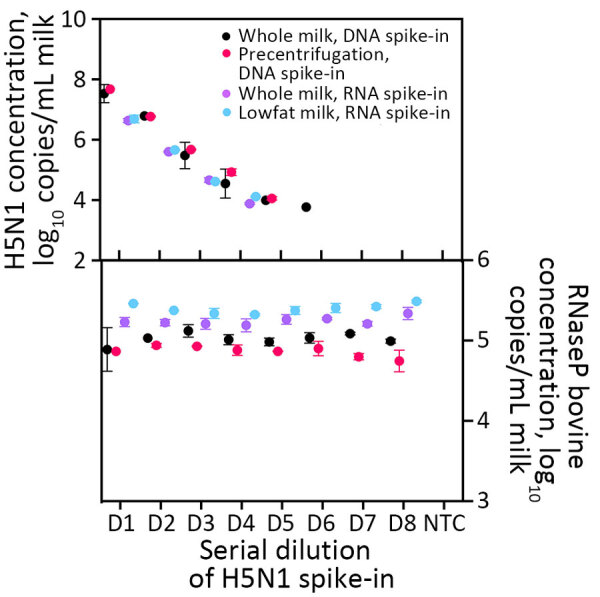
Digital PCR detection of synthetic nucleic acid (top) and RNaseP Bovine (bottom) in study of methods to monitor influenza A(H5N1) virus in dairy cattle milk, Massachusetts, USA. For direct extraction, we extracted 200 μL of milk spiked with serial dilutions of H5N1 synthetic gene fragments. For precentrifugation, we centrifuged samples for 12,000 × *g* for 10 minutes after spike-in, after which we extracted 200 μL. Extractions were performed using the MagMAX CORE extraction kit (Thermo Fisher Scientific, https://www.thermofisher.com).

All 3 extraction kits demonstrated similar recovery of H5N1 virus from milk; the CORE kit exhibited slightly better performance. The CORE (Figure 2) and Prime (Appendix Figure 4) kits showed comparable results in terms of total recovery (down to ≈10^4^ H5N1 virus copies/mL milk) and linearity. Direct nucleic acid extraction from milk was efficient regardless of fat content, with pre-centrifugation offering no increase in viral RNA recovery, in accordance with previous findings (*10*; A. Lail et al., unpub. data, https://www.protocols.io/view/rna-extraction-from-milk-for-hpai-surveillance-n2bvjn6obgk5/v1). In addition, we found no significant difference in detection of H5N1 virus (p = 0.20) or RP_Bov (p = 0.17) using the Wastewater extraction kit on retail milk samples (Appendix Figure 5). We selected the CORE kit for ongoing testing given its low detection limit and slightly better detection of RP_Bov, as well as practical considerations, such as a manufacturer’s protocol for processing milk and kit availability.

**Figure 3:**
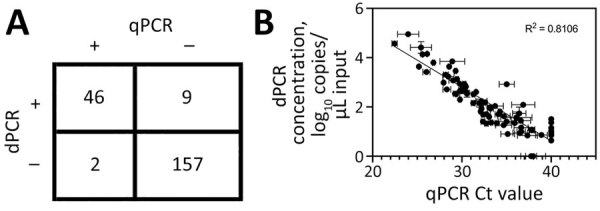
Comparison of dPCR and qPCR virus testing on retail milk samples in study of methods to monitor influenza A(H5N1) virus in dairy cattle milk, Massachusetts, USA. A) Agreement of positive and negative calls of milk samples between the 2 platforms; B) correlation of H5N1 measured by dPCR concentration compared with qPCR Ct value. For plotting purposes, samples not detected by dPCR were graphed with a dPCR concentration of 0 copies/μL, whereas samples not detected by qPCR were graphed with a Ct value of 40. Error bars indicate +1 SD. Ct, cycle threshold; dPCR, digital PCR; qPCR, quantitative PCR; R^2^, coefficient of determination.

To validate protocols on in situ H5N1 virus in milk, we sourced 214 retail milk cartons with diverse characteristics, including fat content and pasteurization processes, from 61 processing plants in 20 states (Table; Appendix Figure 6). Of those, 55 (26%) cartons tested positive for H5N1 RNA by dPCR, whereas 48 (22%) tested positive by qPCR. The platforms gave concordant positive/negative results for 95% (n = 203/214) of samples (Appendix, Figure 7). Nine samples were positive only by dPCR, which could be because of the slightly enhanced LOD of the dPCR assay. Conversely, 2 samples were positive only by qPCR, possibly because of the more stringent thresholding criteria for dPCR. Further, H5N1 RNA dPCR concentrations correlated strongly with qPCR Ct values (R^2^ = 0.81; Figure 3), suggesting the assay is robust on either platform. However, we saw evidence of qPCR standard degradation throughout testing, highlighting the importance of standard material integrity for accurate qPCR quantification. Positive samples were from processing plants in 4 states with reported H5N1 outbreaks (Colorado, Idaho, Michigan, and Texas). We also detected 1 positive sample by both dPCR and qPCR that originated from a processing plant in Missouri, which has not reported H5N1 in cattle. Of note, the location of the processing plant reported on milk containers might or might not correspond to the state in which the milk was initially collected, and this linkage is not publicly available.

**Table:** Breakdown of milk samples tested and their results by processing plant state in study of methods to monitor influenza A(H5N1) virus in dairy cattle milk, Massachusetts, USA

Processing plant state	No. cartons tested	No. positive	Positivity rate, %
AZ	1		
CA	10		
CO*	59	33	56
CT	4		
IA*	9		
ID*	12	5	42
KS*	2		
KY	1		
MA	18		
ME	2		
MI*	14	5	36
MN*	9		
MO	3	1	33
NC*	7		
NH	6		
NY	2		
OH*	3		
TX*	42	13	31
UT	7		
VA	3		
Total	214	57	27

*Indicates state had reported cases of H5N1 in cattle at the time of testing.

**Figure 4:**
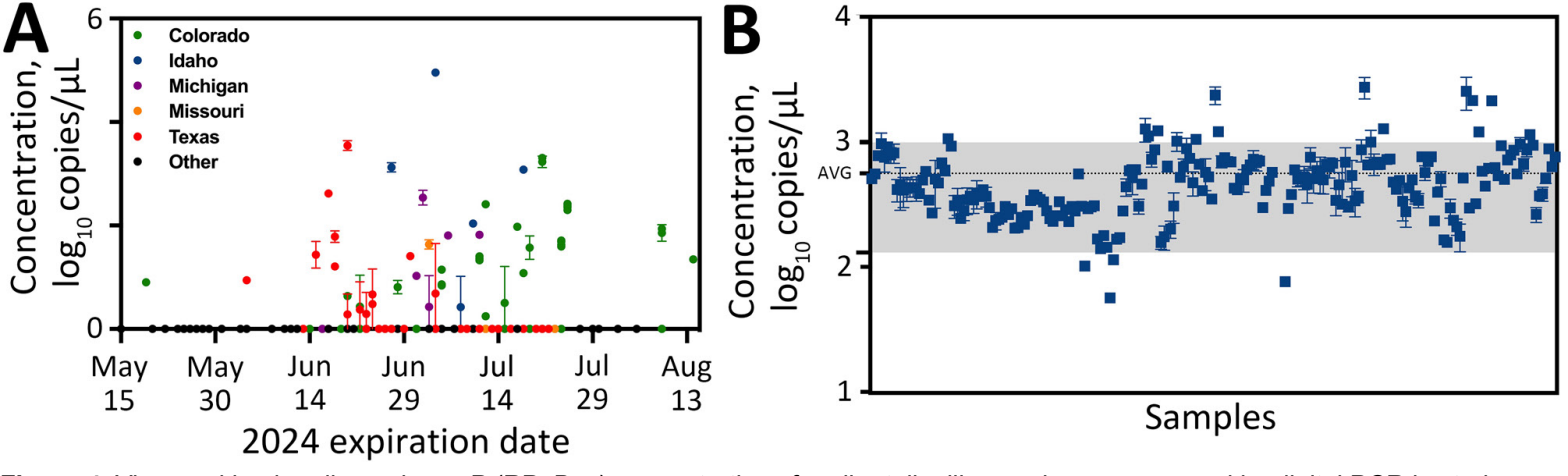
Virus and bovine ribonuclease P (RP_Bov) concentrations for all retail milk samples as measured by digital PCR in study of methods to monitor influenza A(H5N1) virus in dairy cattle milk, Massachusetts, USA. A) Concentration of H5N1 as a function of processing state and expiration date. B) RP_Bov data for all samples. The gray-shaded region corresponds to the average RP_Bov concentration of all data +1 SD. Error bars indicate +1 SD.

We used the RP_Bov assay as an internal sample process control to confirm sample integrity and ensure proper collection and extraction, especially useful to interpret negative H5N1 results. RP_Bov concentrations averaged 560 copies/μL extract (Figure 4); 98% of samples fell within 1 SD. Thus, detection of RP_Bov below ≈100 copies/μL could be effectively used as a measure of milk sample and process integrity.

We next sought to recover genomes from 23 H5N1 virus–positive retail milk samples, testing methods across a range of characteristics including virus concentration, milk type, and pasteurization process. To obtain higher H5N1 virus concentrations for library preparation, we first extracted, pooled, and concentrated 10 samples from each milk container. Ultrapasteurized samples exhibited significantly lower concentration factors than did pasteurized samples as measured by H5N1 copy number (p = 0.015; Appendix Figure 8). Despite being highly concentrated, samples showed no evidence of PCR inhibition by dPCR (p = 0.89; Appendix Figure 9). The recovered RNA content and quality from these samples spanned a wide range as determined by H5N1 copies, total RNA concentration, H5N1 copies per nanogram of RNA, and RNA integrity number score (Appendix Table 7).

**Figure 5:**
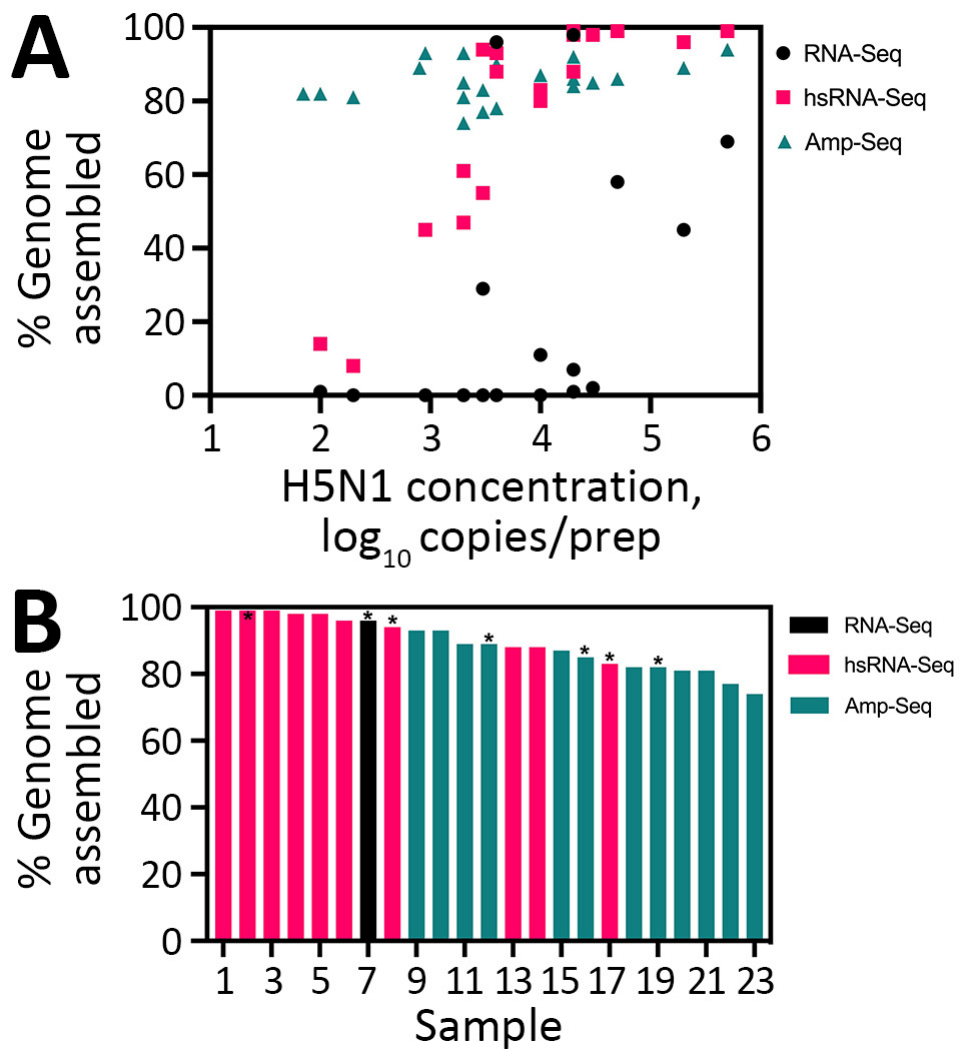
Virus genome assemblies from retail milk samples in study of methods to monitor influenza A(H5N1) virus in dairy cattle milk, Massachusetts, USA. A) Completeness of H5N1 genome assemblies generated by RNA-Seq, virus-enriched (hsRNA-Seq), and targeted H5N1 Amp-Seq as a function of H5N1 copies per milliliter of RNA. B) The most complete H5N1 assembly produced for each sample sorted by length and the underlying sequencing approach. Asterisks (*) above bars indicate ultrapasteurized samples. Amp-Seq, amplicon sequencing; hsRNA-Seq, hybrid-selected metagenomics; RNA-Seq, unbiased metagenomics.

We evaluated 3 library construction methods to assess their efficacy in producing genomes across the range of H5N1 virus concentrations and pasteurization processes: untargeted metagenomic RNA sequencing (RNA-Seq), hybrid-selected RNA-Seq (hsRNA-Seq) enriched for human respiratory viruses including influenza A (albeit not explicitly H5N1) (*11*), and amplicon sequencing (Amp-Seq) of tiled 250-bp H5N1 PCR products (*12*). Despite intense milk preprocessing (such as ultrapasteurization), near-complete (>70% assembly) H5N1 virus genomes were readily recovered from all 23 samples, 12 by hsRNA-Seq (>80%) and 11 by Amp-Seq (>74%). Hybrid selection greatly increased the chances of genome recovery for higher concentration extracts (>500 copies/μL); hsRNA-Seq outperformed RNA-Seq for 11 of 12 samples. At lower concentrations, Amp-Seq resulted in the most complete genomes (Figure 5). Of note, we modified the PCR cycling conditions of a previously reported H5N1 Amp-Seq protocol (*12*), which resulted in improved amplicon generation and genome assemblies (Appendix Figure 10). However, PCR efficiency varied considerably across amplicons; a small fraction of amplicons produced most sequencing reads (Appendix Figure 11).

Phylogenetic analysis showed geographic clustering with other publicly available H5N1 genomes associated with the dairy cattle outbreak (Appendix Figure 12), suggesting the origin of the viruses was consistent with the US state of the processing plant of the milk. Of note, the positive sample originating from Missouri (which has no reports of H5N1 in cattle) clustered with samples from Texas and Michigan, likely pointing to the farm location from which the milk originated, despite being processed in a Missouri plant.

Overall, this study contributes validated methods for the whole workflow from sample to analyzed data for rapid deployment for potential future epidemiologic studies and public health surveillance. On the basis of the methods testing and validation described, we have included a guide to establishing efficient, robust, and scalable H5N1 virus surveillance from bulk milk for implementation in molecular laboratory settings (Appendix). Enabling more laboratories to set up decentralized surveillance will enable us to stay ahead of current and future outbreaks of public concern. The guidelines provided in this article are intended to serve as a blueprint for rapid validation of new molecular detection methods and establishment of surveillance systems for the current H5N1 outbreak and beyond.

This article was originally published as a preprint at https://www.medrxiv.org/content/10.1101/2024.12.04.24318491v1.

Appendix Additional information about establishing methods to monitor influenza A(H5N1) virus in dairy cattle milk, Massachusetts, USA
