# Supplementary material for: Establishing Methods to Monitor Influenza (A)H5N1 Virus in Dairy Cattle Milk, Massachusetts, USA
Source: Emerg Infect Dis. 2025 May;31(Suppl 1):S70–5. doi: 10.3201/eid3113.250087 (PMC12078538; doi:10.3201/eid3113.250087)
Supplement: Appendix — Additional information about establishing methods to monitor influenza A(H5N1) virus in dairy cattle milk, Massachusetts, USA [file 25-0087-Techapp-s1.pdf]

Article DOI: https://doi.org/10.3201/eid3113.250087

EID cannot ensure accessibility for supplementary materials supplied by authors. Readers who have difficulty accessing supplementary content should contact the authors for assistance.

Establishing Methods to Monitor Influenza A(H5N1) Virus in Dairy Cattle Milk, Massachusetts, USA

Appendix

Detailed Methods

Sourcing of retail milk samples and metadata collection

Milk was purchased around greater Boston and obtained from states reported to be impacted by the H5N1 outbreak. To source milk from across the country, milk was purchased using mobile delivery apps by targeting local brands of milk (preference for non-ultra pasteurized bottles) and delivered to a local collaborator. The collaborator then aliquoted the milk into two falcon tubes, sealed in plastic bags, packed on ice or with ice packs, and shipped back to the Broad Institute overnight for processing. Upon receipt, one set of aliquots was stored at −80°C for preservation. The other aliquot proceeded to nucleic acid extraction. In addition, metadata was collected for all milk samples including: brand, type of milk (whole, 2%, etc.), pasteurization process, USDA processing plant code, expiration date, purchase date, and city of purchase.

PCR assay design and characterization

Three PCR primer assays were used in the current study (sequences listed in Table S1). First, an H5N1 assay was designed on a subset of HA sequences from the 2.3.4.4.b clade of H5N1 (referred to as H5_Eva). This assay was only used for initial extraction kit evaluation. In addition, a previously published assay targeting the HA fragment of the H5N1 virus (denoted as H5_Taq) was used in this study (*1*). Finally, an assay was designed to target the Ribonuclease P gene specific to bovine species (RP_Bov) which was used as an internal extraction control. All

digital PCR (dPCR) reactions were run on a Qiacuity One (5plex, Qiagen), using either 24 or 96 well, 8.5k plates and either OneStep Advanced EvaGreen Kit (H5_Eva and RP_Bov assays) or the OneStep Advanced Probe Kit (H5_Taq), following the manufacturer's protocol. Final reaction concentrations of primer and probe for the H5_Taq assay were 400nM and 200nM, respectively, with 5µL of extraction used as template. Final reaction concentration of primers for both the H5_Eva and RP_Bov assay was 500nM, with 1µL of extraction used for extraction kit trials and 2µL of extraction used as template for milk sample testing (RP_Bov only). Every dPCR run included at least one no template control (NTC) that was used to set the threshold, as well as a positive control. The positive control was made by in vitro transcription of an 1800bp synthetic H5N1 DNA sequence of the HA segment to make an RNA standard. Transcribed RNA was purified using RNAClean XP beads (Beckman Coulter) following manufacturer recommendations. Purified transcribed RNA was then quantified using the Invitrogen Qubit RNA High Sensitivity (HS) kit following the manufacturer protocol and refined by dPCR. Cycling and imaging protocols for all assays can be found in Appendix Tables 3,4. After thresholding to the NTC, a sample had to have at least three positive partitions to be considered positive (*1*,*2*). Concentrations of samples were then calculated based on dPCR reported concentration, input volume of extraction, and dilution factor.

All quantitative PCR (qPCR) assays were conducted on a QuantStudio 6 Flex (ThermoFisher) using TaqMan RNA-to-C_t 1-Step Kit (ThermoFisher), following manufacturer's recommendations. All samples were run on qPCR in triplicate, 10µL reactions with the final concentration of H5 primers and probe at 500nM and 250nM, respectively (optimization data and cycling conditions can be found in Appendix). For milk samples, 1µL of extract was used as a template. A sample was considered positive if 2 out of 3 replicates amplified, and if at least one of the extraction replicates was positive. The amplified Ct values were then averaged for subsequent analysis.

PCR assay performance was evaluated using synthetic RNA of the HA segment of the H5N1 virus. The limit of detection (LOD_{90}) was defined as the lowest concentration of target copies detected in at least 18/20 replicates. Linearity was evaluated from serial dilutions over the range of detection for both PCR platforms. Finally, the standard curves of all qPCR runs were evaluated for overall assay efficiency.

Extraction kit evaluation

Three commercially available extraction kits were evaluated for their potential to recover nucleic acid from a milk matrix. All kits chosen were bead-based and high-throughput kits compatible with the KingFisher Flex instrument (ThermoFisher) and evaluated for performance by dPCR targeting H5N1 (H5_Eva) and the Ribonuclease P gene of bovines (RP_Bov). First, the MagMAX Prime kit was tested by spiking serial dilutions (10^2 - 10^8 copies/mL) of an 1800bp synthetic DNA fragment of the HA sequence of the H5N1. To evaluate the effect of the milk matrix on recovery, both whole and low-fat milk were tested and diluted with phosphate-buffered saline (PBS) so that the milk matrix was present at 25%–100% before being spiked with target. In addition, two pre-centrifugation conditions (either 12000xg for 10 minutes or 1200xg for 30 minutes) were tested with the 100% whole milk condition. Next, the MagMAX CORE kit was evaluated following the manufacturer's "Simple Workflow" which specifies milk as an input type. For this experiment, whole milk was spiked with a dilution series of H5N1 synthetic DNA fragments (10^2 - 10^8 copies/mL) and either processed directly or pre-centrifuged at 12000xg for 10 minutes. Finally, the CORE kit was tested with serial dilutions (10^2 - 10^8 copies/mL) of H5N1 synthetic RNA fragments spiked into both whole and low-fat milk and measured by the H5_Taq assay.

Finally, the MagMAX Wastewater kit was evaluated head-to-head with the MagMAX CORE kit on a subset of retail milk samples previously identified as positive through evaluation with the CORE kit. For this comparison, all samples were re-extracted with the CORE kit as well as processed with the Wastewater kit on the same day. Both kits were used following the manufacturer's instructions, using 200µL of milk as input into extraction.

Nucleic acid isolation of retail milk samples

All milk samples were extracted in duplicate using the MagMAX CORE kit on a KingFisher Flex following the manufacturer's "Simple Workflow" for 200µL of milk input. A subset of positive samples were chosen to be evaluated by sequencing. To obtain enough RNA for sequencing, these samples were re-extracted by the CORE kit 10 times and subsequently concentrated using the RNA-Clean and Concentrator Kit (Zymo) using manufacturer protocols, including on column DNase treatment and adjusting the amount of binding buffer and ethanol to match the total elution volume and passing the entire volume in multiple loading steps through the column. Effects of inhibition of concentrated samples were evaluated by the dPCR H5_Taq

assay spiking in two different volumes of template (1µL vs 2µL of input) and calculating total concentration per µL of extract.

Surveillance of raw milk samples from Massachusetts farms

Bulk tank samples from all Massachusetts cattle dairy farms (n = 95) were collected by Massachusetts Department of Agricultural Resources (MDAR) employees on a monthly basis starting on August 6, 2024 and delivered to the Broad Institute for processing. Samples were collected in 2 ounce plastic containers, typical for bulk tank sampling, and transported on ice to the lab for testing. Samples were immediately pasteurized onsite in a heated water bath by ensuring the internal temperature of the collection bottle reached 72°C for at least 15 seconds per USDA protocols (*3*). The samples were immediately placed on ice to cool and then proceeded through the same workflow as described above for nucleic acid extraction and subsequent dPCR analysis.

Preparation of sequencing libraries

RNA samples for sequencing were run on an RNA 6000 Pico Bioanalyzer chip (Agilent) to determine total RNA concentration, size distribution, and RNA Integrity (RIN) scores. Water and a water extraction control served as blank negative controls for library construction.

Unbiased metagenomic libraries (RNA-Seq). RNA-Seq libraries were generated by the xGen RNA library prep kit (IDT) with 8-base UDI Primers Plate 1 (IDT). Input RNA volumes were adjusted to not exceed 125ng RNA. Time and temperature of the RNA fragmentation step were modified depending on the RIN score following the manufacturer's guidance for low-quality RNA. RNA-Seq libraries were amplified by 5 (>100ng input RNA) or 8 (<100ng input RNA) PCR cycles.

Hybrid selected RNA-Seq libraries (hsRNA-Seq). xGen RNA-Seq libraries were pooled in groups of 2–4 libraries (400–900 ng total per pool) from RNA samples with similar (i.e., same order of magnitude) H5N1 copies/µL. Hybrid selection was performed using the Respiratory Virus Research Capture panel as bait (Twist Biosciences) with the Target Enrichment Standard Hybridization v2 kit (Twist Biosciences) following the manufacturer's protocols. Based on initial trials to determine the minimum number of PCR cycles necessary, libraries for hybrid selection were amplified with 9 or 12 PCR cycles to generate >100ng input RNA. Preparative post-hybrid selection PCR reactions (25µL bead slurry in 100µL 1x HiFi

HotStart ReadyMix (Roche)) containing 4µM Illumina P7 and 4µM Illumina P5 primers were run for 12–16 cycles for this study.

H5N1 Amp-Seq libraries (Amp-Seq). We used 5µL of RNA as input to generate Amp-Seq libraries irrespective of concentration and H5N1 content. cDNA was generated in 20µL reactions using the Superscript IV kit (Thermofisher) following the manufacturer's protocols. Two subsequent PCR reactions (25µL each) were comprised of 1x HiFi HotStart ReadyMix (Roche), 4µL cDNA, and 1.6 µM of previously developed primer pool 1 or 2 specific to H5N1 (*4*). We used a modified thermoprofile from the original published protocol, namely shortening the annealing step and adding an extension step (Details can be found in Table S6).

PCR products were purified with 0.8 volumes of AmPure XP beads (Beckman Coulter) at 0.8 volumes for RNA-Seq and hsRNA-Seq or 1.5 volumes for Amp-Seq, quantitated by Qubit DNA High Sensitivity kit (Thermo Fisher), and characterized on a dsDNA High Sensitivity Bioanalyzer chip (Agilent) using 1ng of PCR product as input. For Amp-Seq, primer pool 1 and 2 PCR products were combined and Illumina sequencing libraries were generated using scaled-down half-reactions of the NEBNext Ultra II DNA Library Prep Kit with multiplex oligos (New England Biolabs), adjusting the adaptor dilution to the combined input DNA amounts as recommended by the manufacturer: 1:25 for <5ng; 1:12 for 5–25ng; 1:6 for 25–100ng and no dilution for >100ng) and a simple clean-up with 0.9 volumes AmPure beads before the final PCR amplification (6–10 cycles).

Pooled sequencing libraries were sequenced with paired-end 151-base reads on 300-cycle NextSeq 2000 cartridges (Illumina). Separate sequencing runs of metagenomic or HS_metagenomic libraries contained a 10% PhiX spike-in. Four NextSeq sequencing runs were conducted in total: one with 12 metagenomic libraries (0.65nM), one with a hybrid capture of the aforementioned 12 libraries (0.65nM loading concentration), one with the two amplicon sequencing approaches (0.65nM), and one with a superpool of all of the above methodologies at molar ratios of 0.16 (Amp-Seq): 0.42 (metagenomic): 0.42 (HS_metagenomic) for sequencing.

Genomic analysis

Basecalling and demultiplexing. NextSeq sequencing runs (151bp paired end, with 8bp dual barcodes) were basecalled and demultiplexed using Picard using custom specified read structures to accommodate for the xGen library protocol. The first two sequencing runs (RNA-

Seq and hsRNA-Seq libraries) were demultiplexed using read structure 151T8B8B20S131T to skip the first 20 bases of the 2nd read that contains artificial sequences added during the "adaptase" step of the protocol. The last two sequencing runs (including Amp-Seq) were demultiplexed using read structure 34S117T8B8B34S117T to remove all PCR primers from the AVRL H5N1 protocol (the longest primer in that design is 34bp, and they appear at the beginning of each read). This produces hard-trimmed reads containing only target sequences and obviates the need for any post-alignment based trimming during consensus sequence generation.

Genome assembly. For each sequencing library, consensus influenza genomes were produced using a standard consensus generation pipeline used previously for Ebola, Zika and SARS-CoV-2 genomes (*5–8*). The H5N1 Bovine/texas/24–029328–01/2024 reference genome (PP599462.1 through PP599469.1) was used as the reference for all assemblies. For RNA-Seq and hsRNA-Seq libraries, default parameters for assemble_refbased were used. For Amp-Seq libraries, the min_coverage parameter was increased to 20 (from default 3; as the reads are non-independent) and skip_mark_dupes was set to true (from default false; we skip PCR duplicate removal since all reads are PCR duplicates), similar to previously established methods[37]. As all reads were hard-trimmed of primers during basecalling, we did not perform any post-alignment trimming with BED files. For each sample sequenced by multiple methods, we used the hsRNA-Seq genome if it recovered at least 75% of the genome, and, if not, used the Amp-Seq genome if it recovered at least 75% of the genome, and, if neither was above 75%, declared the sample unsuccessful.

Phylogenetic analysis. Phylogenetic analysis was performed by releasing successful genomes on NCBI Genbank, allowing it to be automatically incorporated into the Moncla Lab / Nextstrain avian-flu builds for the cattle-associated outbreak. Briefly, this utilizes concatenated genomes for the build (all eight segments combined into a pseudo-chromosome) due to the negligible effects of reassortment at the outbreak timescale, excludes certain outlier genomes, and imposes other build-specific parameters found at https://github.com/nextstrain/avian-flu?tab = readme-ov-file#h5n1-cattle-outbreak-2024. The resulting build can be found at https://nextstrain.org/avian-flu/h5n1-cattle-outbreak/genome?c = division&d = tree,entropy&f_host = Cattle&f_submitting_lab = Broad%20Institute%20Genomic%20Center%20for%20Infectious%20Diseases,%20Genomic%20Center%20for%20Infectious%20Diseases&m = num_date&p = full.

Data Availability. Sequence data are available at NCBI/INSDC under BioProject PRJNA1134696. This includes BioSamples described under the One Health Enterics package, SRA records for each sequencing replicate and method described above, and Genbank & Assembly records for at most one genome per sample, selected by the criteria described above.

Statistical analyses

All statistical analysis was completed in GraphPad Prism with statistical significance defined as $p < 0.05$. Correlation between dPCR concentration and qPCR Ct value as well as tests of linearity were fit by simple linear regression. One-way ANOVA was used to compare effects of different conditions when multiple conditions were compared whereas a paired t test was used when two conditions were being compared at once.

Comparison of dPCR and qPCR technologies

Digital PCR (dPCR) has become more widely adopted in recent years, particularly owing to its adoption by the wastewater-based epidemiology field as the technology of choice for surveillance of SARS-CoV-2 (*9*). This has increased the literature base for the platform and increased confidence in the technology. In particular, dPCR excels in enhanced sensitivity (*9*–*13*), absolute quantification without reliance on positive standard material (*14*), robustness to PCR inhibitors (*14*,*15*), and decreased interlaboratory variation (*16*). In contrast, quantitative PCR (qPCR) remains the preferred technology in clinical settings, as evidenced by the majority of data generation from individual patients during the SARS-CoV-2 pandemic (*17*), despite dPCR demonstrating superior performance in many clinical studies (*11*,*14*). Benefits of qPCR include a broader dynamic range of detection, higher throughput capabilities, lower cost per sample, and more common lab availability (*9*,*12*). However, qPCR has been shown to be commonly affected by PCR inhibitors and provides unreliable quantification (*14*,*18*), especially since it requires a well characterized positive control material for accurate quantification of target (*10*,*19*). In cases where qPCR is used, dPCR can be useful for quantification of standards to ensure quality of materials (*14*,*20*) to mitigate the effect of qPCR standard degradation and inaccuracy.

Suggested guidelines for setting up laboratory testing capacity for H5N1 in milk

To stay ahead of the current outbreak, as well as to prepare for worsening conditions such as human-to-human spread, we encourage labs across the nation to set up capacity for H5N1 testing. Based on the study findings, we are able to provide some recommendations to help jumpstart detection at any molecular testing lab by following the provided guidelines:

1. Select PCR detection platform

a. Important considerations are equipment availability, throughput requirements, and sensitivity requirements.

2. Order materials

a. Order the H5_Taq primers and probes as well as the RP_Bov primers. If using a different RT-PCR kit than used in the present study, be careful to adjust cycling conditions based on the specific kit characteristics.

b. Order a nucleic acid extraction kit compatible with your available lab equipment. Several other extraction kits may be compatible than the ones validated in the present study but each should first be evaluated individually before milk sample surveillance.

3. PCR assay set up and validation

a. Synthetic gene fragments can be ordered from companies such as Twist Biosciences or IDT. Order the gene fragments with the T7 polymerase promoter site on the 5′ end to be able to transcribe into RNA synthetic fragments.

b. In vitro transcribe the gene fragments to create high titer RNA synthetic standards. Clean up material using AmPure XP beads and quantify using Qubit to dilute to the appropriate concentration. A helpful tool to calculate copy numbers is available at: https://nebiocalculator.neb.com/

c. Verify and refine standard material concentration by dPCR, if available.

d. Create a serial dilution of standard material (ideally from 1E1–1E7 copies/uL for qPCR and between 1E1–1E4 for dPCR).

e. Verify PCR performance on the standard material dilutions.

i. Ensure acceptable performance metrics are met:

1. Linearity >90%,

2. qPCR efficiency between 90%–110%

3. LOD of 10 copies/μL extract or lower

4. Extraction kit set up and validation

a. Create contrived positive milk samples by spiking negative milk with a serial dilution of synthetic gene fragments (Ideally test a range of concentrations such as 1E2 - 1E8 copies/mL).

b. Extract contrived samples according to kit specifications.

c. Evaluate recovered copy number by PCR.

d. Ensure acceptable performance metrics are met:

i. Linearity >90%,

ii. Process LOD $\lesssim 10^4$ copies/mL of milk

iii. Consistent RP_Bov detection across H5 dilutions.

5. Sourcing positive milk samples and further validation of lab methods

a. Identify current outbreak locations at: https://www.aphis.usda.gov/livestock-poultry-disease/avian/avian-influenza/hpai-detections/hpai-confirmed-cases-livestock

b. Identify state processing plant codes at: www.whereismymilkfrom.com

c. Purchase milk at local grocery stores originating from states with active outbreaks. As well, source milk from collaborators living in the same geographic regions as active outbreaks to increase your chances of obtaining positive milk samples.

6. Raw milk surveillance

a. First, verify the lab is capable of receiving and processing raw (unpasteurized) milk and that this does not violate current biosafety protocols.

b. It is suggested to pasteurize raw milk on site before processing to limit worker exposures while retaining genomic material.

i. Samples can be pasteurized by heating to an internal temperature of 72°C for at least 15 seconds per the USDA protocol (*3*). It is recommended to validate the temperature and holding time for the specific sampling containers that will be received using retail milk before handling raw milk.

ii. After heating, cool the milk samples on ice before processing through the same workflow for commercially pasteurized milk.

7. Sequencing and Data Reporting

a. For low concentration samples (below 500 H5N1 copies/uL extract), optionally re-extract positive samples up to 10 times. Concentrate extracts using a commercially available kit such as the Zymo Clean and Concentrator Kit or another preferred method that includes DNaseI digestion to remove DNA.

b. For samples with >500 H5N1 copies/uL, hybrid-selected metagenomic sequencing is suggested to result in more complete genome assemblies. For samples <500 H5N1 copies/uL, we suggest using an Amp-Seq protocol, such as the one used in the current study.

c. Deposit raw sequencing reads to GenBank as well as submit assembled genomes to NextStrain.

8. Large Scale Testing Considerations

a. Depending on scope of the outbreak and number of samples to be processed, consider if a partner lab is needed for scaled up testing.

b. Partner labs could allow for rapid transition of testing based on needs.

References

1. Wolfe MK, Duong D, Shelden B, Chan EMG, Chan-Herur V, Hilton S, et al. Detection of Hemagglutinin H5 Influenza A Virus Sequence in Municipal Wastewater Solids at Wastewater Treatment Plants with Increases in Influenza A in Spring, 2024. ES&T Letters. 2024 May 20;11(6), 526–532.

2. Huisman JS, Scire J, Caduff L, Fernandez-Cassi X, Ganesanandamoorthy P, Kull A, et al. Wastewater-Based Estimation of the Effective Reproductive Number of SARS-CoV-2. Environ Health Perspect. 2022;130:57011. PubMed https://doi.org/10.1289/EHP10050

3. Food and Drug Administration, Department of Health and Human Services. Code of Federal Regulations: Part 131–Milk and Cream. 1977 Mar 15. https://www.ecfr.gov/current/title-21/chapter-I/subchapter-B/part-131.

4. Vuyk WC, Lail A, Emmen I, Hassa N, Tiburcio PB, Newman C, et al. Whole genome sequencing of H5N1 from dairy products with tiled 250bp amplicons v1. 2024 Jun 5. protocols.io. dx.doi.org/10.17504/protocols.io.kqdg322kpv25/v1. https://doi.org/10.17504/protocols.io.kqdg322kpv25/v1

5. Holmes EC, Dudas G, Rambaut A, Andersen KG. The evolution of Ebola virus: Insights from the 2013-2016 epidemic. Nature. 2016;538:193–200. PubMed https://doi.org/10.1038/nature19790

6. Lemieux JE, Siddle KJ, Shaw BM, Loreth C, Schaffner SF, Gladden-Young A, et al. Phylogenetic analysis of SARS-CoV-2 in Boston highlights the impact of superspreading events. Science. 2021;371:eabe3261. PubMed https://doi.org/10.1126/science.abe3261

7. Wohl S, Metsky HC, Schaffner SF, Piantadosi A, Burns M, Lewnard JA, et al. Combining genomics and epidemiology to track mumps virus transmission in the United States. PLoS Biol. 2020;18:e3000611. PubMed https://doi.org/10.1371/journal.pbio.3000611

8. Park D, Tomkins-Tinch C, Ye S, Jungreis I, Arenas FN, Shlyakhter I, et al. broadinstitute/viral-pipelines: v2.3.6.0. Zenodo. 2024 Nov.

9. Tiwari A, Ahmed W, Oikarinen S, Sherchan SP, Heikinheimo A, Jiang G, et al. Application of digital PCR for public health-related water quality monitoring. Sci Total Environ. 2022;837:155663. PubMed https://doi.org/10.1016/j.scitotenv.2022.155663

10. Ahmed W, Simpson SL, Bertsch PM, Bibby K, Bivins A, Blackall LL, et al. Minimizing errors in RT-PCR detection and quantification of SARS-CoV-2 RNA for wastewater surveillance. Sci Total Environ. 2022;805:149877. PubMed https://doi.org/10.1016/j.scitotenv.2021.149877

11. Liu C, Shi Q, Peng M, Lu R, Li H, Cai Y, et al. Evaluation of droplet digital PCR for quantification of SARS-CoV-2 Virus in discharged COVID-19 patients. Aging (Albany NY). 2020;12:20997–1003. PubMed https://doi.org/10.18632/aging.104020

12. Park S, Rana A, Sung W, Munir M. Competitiveness of Quantitative Polymerase Chain Reaction (qPCR) and Droplet Digital Polymerase Chain Reaction (ddPCR) Technologies, with a Particular Focus on Detection of Antibiotic Resistance Genes (ARGs). Appl Microbiol. 2021;1:426–44. https://doi.org/10.3390/applmicrobiol1030028

13. Ahmed W, Smith WJM, Metcalfe S, Jackson G, Choi PM, Morrison M, et al. Comparison of RT-qPCR and RT-dPCR Platforms for the Trace Detection of SARS-CoV-2 RNA in Wastewater. ACS ES T Water. 2022;2:1871–80. 10.1021/acsestwater.1c00387 PubMed https://doi.org/10.1021/acsestwater.1c00387

14. Kuypers J, Jerome KR. Applications of Digital PCR for Clinical Microbiology. J Clin Microbiol. 2017;55:1621–8. PubMed https://doi.org/10.1128/JCM.00211-17

15. Hinkle A, Greenwald HD, Metzger M, Thornton M, Kennedy LC, Loomis K, et al. Comparison of RT-qPCR and digital PCR methods for wastewater-based testing of SARS-CoV-2. unpub. data

16. Boxman ILA, Molin R, Persson S, Juréus A, Jansen CCC, Sosef NP, et al. An international inter-laboratory study to compare digital PCR with ISO standardized qPCR assays for the detection of norovirus GI and GII in oyster tissue. Food Microbiol. 2024;120:104478. PubMed https://doi.org/10.1016/j.fm.2024.104478

17. Nyaruaba R, Mwaliko C, Dobnik D, Neužil P, Amoth P, Mwau M, et al. Digital PCR Applications in the SARS-CoV-2/COVID-19 Era: a Roadmap for Future Outbreaks. Clin Microbiol Rev. 2022;35:e0016821. PubMed https://doi.org/10.1128/cmr.00168-21

18. Bivins A, Kaya D, Bibby K, Simpson SL, Bustin SA, Shanks OC, et al. Variability in RT-qPCR assay parameters indicates unreliable SARS-CoV-2 RNA quantification for wastewater surveillance. Water Res. 2021;203:117516. PubMed https://doi.org/10.1016/j.watres.2021.117516

19. Taylor SC, Laperriere G, Germain H. Droplet Digital PCR versus qPCR for gene expression analysis with low abundant targets: from variable nonsense to publication quality data. Sci Rep. 2017;7:2409. PubMed https://doi.org/10.1038/s41598-017-02217-x

20. Park C, Lee J, Hassan ZU, Ku KB, Kim SJ, Kim HG, et al. Comparison of Digital PCR and Quantitative PCR with Various SARS-CoV-2 Primer-Probe Sets. J Microbiol Biotechnol. 2021;31:358–67. PubMed https://doi.org/10.4014/jmb.2009.09006

Appendix Table 1. PCR primer sequences used in this study.

Primer	Sequence (5′ >3′)	Amplicon length (bp)	Annealing temp	Reference
H5_Eva_F	TGCAAACAATTCGACAGAGC	98	58	This Study
H5_Eva_R	GCTTCCCGTTGTGTGTTTTT			
H5_Taq_F	TATAGARGGAGGATGGCAGG	171	59	Wolfe et al.
H5_Taq_R	ACDGCCTCAAAYTGAGTGTT			
H5_Taq_P	(FAM)AGGGGAGTGGKTACGCTGCRGAC(IBFQ)			
RP_Bov_F	CGATTTGGACCTGCGGGCG	65	58	This Study
RP_Bov_R	GAGCAGCGTTCTCCACGAGC			

Appendix Table 2. Gene fragment sequences used as positive control, including T7 priming sequence.

Name	Sequence (5′ >3′)
H5N1_gblock	gaaatTAATACGACTCACTATAgggACCAGAGGTTGGCACCAAAAATAGCTACTAGATCCCAAGTAAACGGCA<br>ACGTGGAAGAATGGACTTCTTCTGGACAATCTTAAAACCAGATGATGCAATCCATTTCGAGAGTAATGGAAA<br>TTTCATTGCTCCAGAATATGCATACAAAATTGTCAAGAAAGGGGACTCAACAATTATGAAAAGTGGAGTGGA<br>ATATGGCCACTGCAACACCAAATGTCAAACCCCAGTAGGTGCGATAAATTCTAGTATGCCATTCCACAACAT<br>ACATCCTCTCACCATTGGGGAATGCCCCAAATACGTGAAGTCAAACAAGTTGGTCCTTGCGACTGGGCTCA<br>GAAATAGTCCTCTAAGAGAAAAGAGAAGAAAAAGAGGCCTGTTTGGGGCGATAGCAGGGTTTATAGAGGGA<br>GGATGGCAGGGAATGGTTGATGGTTGGTATGGGTACCATCATAGCAATGAGCAGGGGAGTGGGTACGCTG<br>CGGACAAAGAATCCACCCAAAAGGCAATAGATGGAGTTACCAATAAGGTCAACTCAATCATTGACAAAATGA<br>ACACTCAATTTGAGGCAGTTGGAAGGGAGTTTAATAACTTAGAAAGGAGGATAGAGAATTTGAACAAGAAAA<br>TGGAAGACGGATTCCTAGATGTCTGGACCTATAATGCTGAACTTCTAGTTCTCATGGAAAACGAGAGGACTC<br>TAGATTTCCATGATTCAAATGTCAAGAACCTTTACGACAAAGTCAGATTACAGCTTAGGGATAATGCAAAGG<br>AGCTGGGTAACGGCTGTTTCGAATTCTATCACAGATGTGATAATGAATGTATGGAAAGTGTGAGAAATGGGA<br>CGTATGACTACCCTCAGTATTCAGAAGAAGCAAGATTAAAAAGAGAAGAAATAAGCGGAGTGAAATTAGAAT<br>CAGTAGGAACTTACCAGATACTGTCAATTTATTCAACAGCGGCAAGTTCCCTAGCACTGGCAATCATGATGG<br>CTGGTCTATCTTTATGGATGTGCTCCAATGGGTCGTTACAGTGCAGAATTTGCATTTAGAAAACACCCTTGTT<br>TCTACTAGTATGCATCTCGT
RP_Bov_gblock	gaaatTAATACGACTCACTATAgggGACTTCAGCATGGCGGTGTTTGCCGATTTGGACCTGCGGGCGGTTCT<br>GACCTGAAGGCGCTGCGTGGGCTCGTGGAGAACGCTGCTCACCGTGAGTCTCCCGGGCTTCGCGGGCCC<br>CCGTGCCTGCGCTGTCTCGCTGTCCCTAGGCTGTAGAGCCATGCTCTGGAGAGACCCGGCGGGCCTAGTT<br>CTGGTGTCCTGGGGCCTCCGGCGTGTCCTTGGAAACTGATGCCCCTGCGGTGTTGCTCTGACCCGCGGGA<br>AACTCGAAAGCACTGGGGAGACGTTACCCAGTCCAGCTCCTTCTGTCTTGGGAATTGAGGAAACTGAGGCC<br>CTGACATGGCGGGGGATCTGCATGGGCCTCACAGCTGATGGATAGAAGAAAACAGGGCTCCCAAGTGTAC<br>ACCTACACGAGTGCTT

Appendix Table 3. dPCR cycling conditions for H5_Taq

Step	Temp (°C)	Time	Cycles
RT	50	40m	1
RT inactivation	95	2m	1
Denaturation	95	5s	40
Annealing	59	30s	
Imaging	500ms exposure, 6 gain		

Appendix Table 4. dPCR cycling conditions for H5_Eva and RP_Bov run as EvaGreen assays.

Step	Temp (°C)	Time	Cycles
RT	50	40m	1
RT Inactivation	95	2m	1
Denaturation	95	15s	40
Annealing	58	15s	
Extension	72	15s	
Cooling	40	5m	1
Imaging	200ms exposure, 3 gain		

Appendix Table 5. qPCR cycling conditions for H5_Taq

Step	Temp (°C)	Time	Cycles
RT	48	15m	1
RT inactivation	95	10m	1
Denaturation	95	15s	40
Annealing	59	1m	

Appendix Table 6. PCR cycling conditions for generating Amp-Seq libraries. Of note, this includes a shorter annealing step (shortened from 5 min) and the addition of an extension step compared to the originally developed protocol.

Step	Temp (°C)	Time	Cycles
Activation	98	1m	1
Denaturation	98	15s	35
Annealing	65	30s	
Extension	72	45s	
Final Extension	72	2m	1

Appendix Table 7. Input RNA samples for sequencing and best genome assemblies for them*

Sample	Sample name	State	Pasteurization	H5N1 copies/μL	Total RNA ng/μL	Copies H5N1 per ng RNA	RIN	Longest assembly (kb)	% of Genome	Sequencing method
1	ME015	ID	P	90001	12.81	7026	2	13526	99.20%	hsRNA-Seq
2	ME023	ID	UP	4345	0.29	14780	2.4	13520	99.20%	hsRNA-Seq
3	MF006	ID	P	6253	1.04	6007	2.8	13476	98.90%	hsRNA-Seq
4	MF014	CO	P	14173	67.41	210	5.5	13334	97.80%	hsRNA-Seq

Sample	Sample name	State	Pasteurization	H5N1 copies/µL	Total RNA ng/µL	Copies H5N1 per ng RNA	RIN	Longest assembly (kb)	% of Genome	Sequencing method
5	MF013	CO	P	13492	58.3	231	5.8	13311	97.70%	hsRNA-Seq
6	MD034	TX	P	37574	8.02	4686	5.4	13152	96.50%	hsRNA-Seq
7	ME001	CO	UP	857	1.27	677	2.5	13050	95.70%	RNA-Seq
8	MD050	CO	UP	518	2.6	199	2.2	12850	94.30%	hsRNA-Seq
9	MD027	TX	P	344	43.66	8	3.5	12741	93.50%	Amp-Seq
10*	ME020	ID	P	407	0.1	4157	2.1	12619	92.60%	Amp-Seq
11*	ME034	TX	P	155	0.24	650	2.4	12110	88.80%	Amp-Seq
12*	MD031	MO	UP	145	1.02	142	2.5	12065	88.50%	Amp-Seq
13	MF011	CO	P	327	1.56	210	6	12044	88.40%	hsRNA-Seq
14	MD035	TX	P	4329	8.27	524	5.4	12009	88.10%	hsRNA-Seq
15	ME013	MI	P	2911	8.77	332	7.2	11867	87.10%	Amp-Seq
16*	MD041	CO	UP	333	1.45	229	2.3	11614	85.20%	Amp-Seq
17	MC023	CO	UP	2623	10.48	250	2	11277	82.70%	hsRNA-Seq
18	ME018	ID	P	28	0.82	34	2.1	11211	82.20%	Amp-Seq
19*	ME003	CO	UP	14	1.6	9	2.4	11181	82.00%	Amp-Seq
20	MD029	TX	P	968	42.73	23	3.2	11086	81.30%	Amp-Seq
21	MF016	CO	P	86	49.36	2	6.3	11024	80.90%	Amp-Seq
22	MF021	CO	P	870	34.62	25	6.8	10456	76.70%	Amp-Seq
23	ME010	MI	P	663	41.55	16	2.3	10144	74.40%	Amp-Seq

*Samples are in the same order as in Figure 5B, i.e., ranked by the length of the most complete genome assembly. H5N1 copies/ul RNA was determined by dPCR. Total RNA concentration and RIN score were determined electrophoretically on a BioAnalyzer chip. We note that the RIN scoring of this assay is for eukaryotic RNA samples, but the length of prominent rRNA bands suggest that much of the RNA is bacterial. Amp-Seq data were generated for all 23 samples. RNA-Seq or hsRNA-Seq data were generated for 18 samples. No RNA-Seq or hsRNA-Seq was performed on the 5 samples indicated by an asterisk.

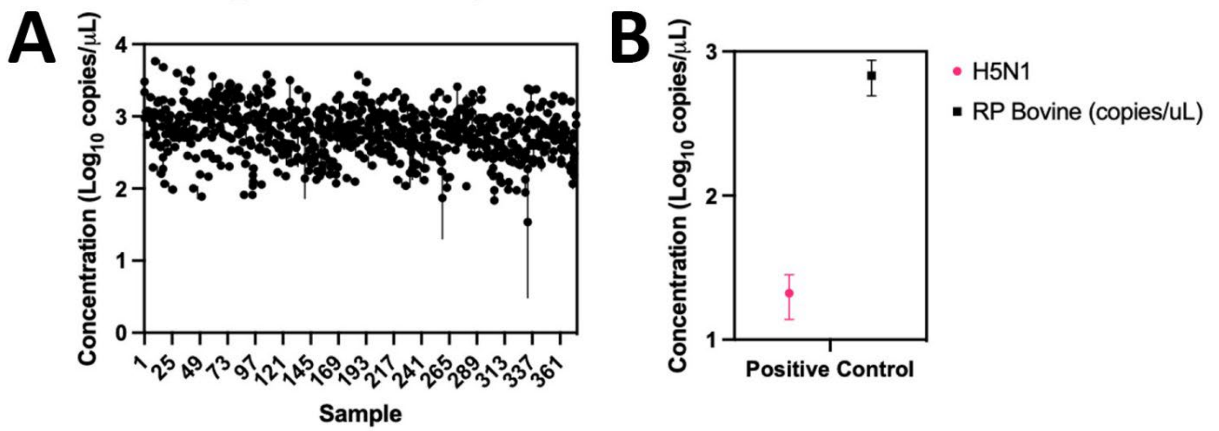

Appendix Figure 1. A) Concentration of RnaseP Bovine (RP_Bov) in samples from Massachusetts farms. Note: all samples were negative for presence of H5N1. B) Summary data from positive control milk samples run along with farm samples. Positive control samples were aliquoted from known positive commercial milk samples.

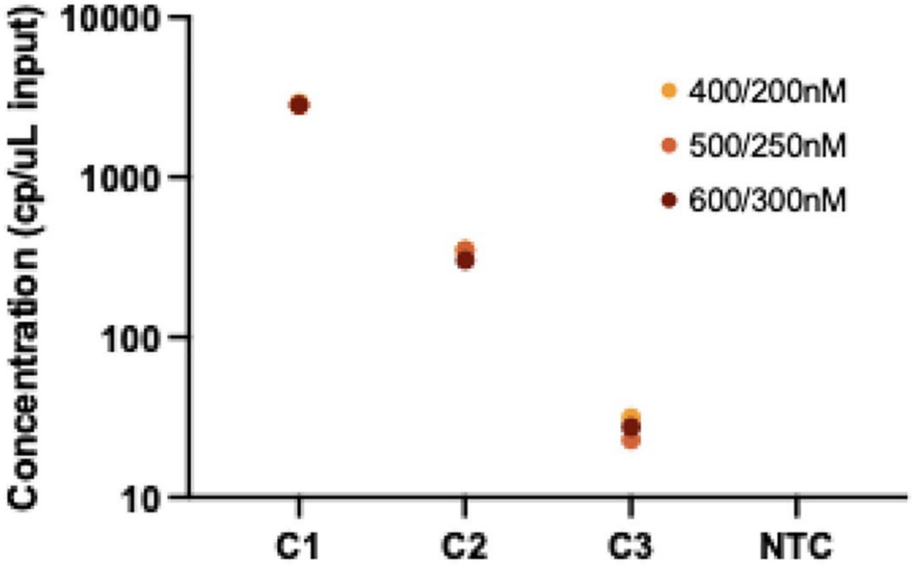

Appendix Figure 2. Optimization of primer and probe concentrations for H5_Taq dPCR assay using synthetic nucleic acid targets over three orders of magnitude (C1-C3). Based on this, 400/200nM final primer/probe concentration was selected.

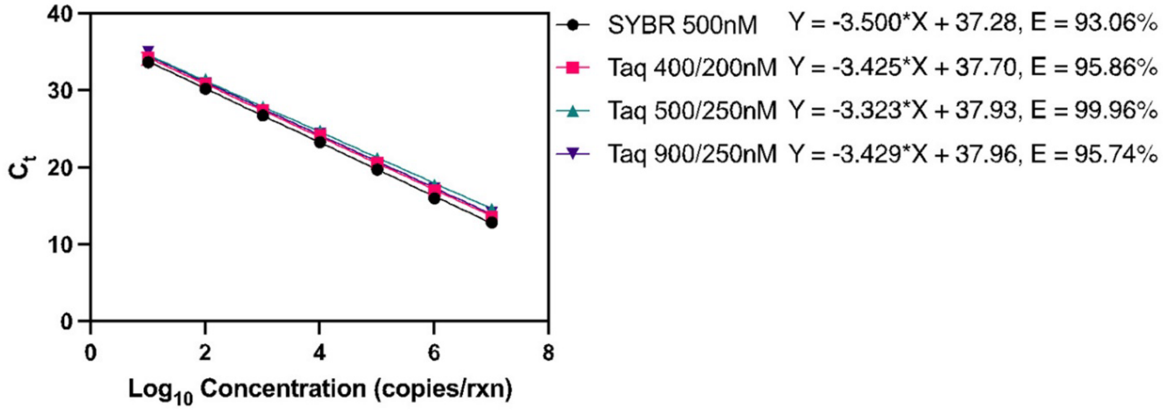

Appendix Figure 3. Optimization of primer and probe concentrations for H5_Taq qPCR assay. Based on this, 500/250nM final primer/probe concentration was selected.

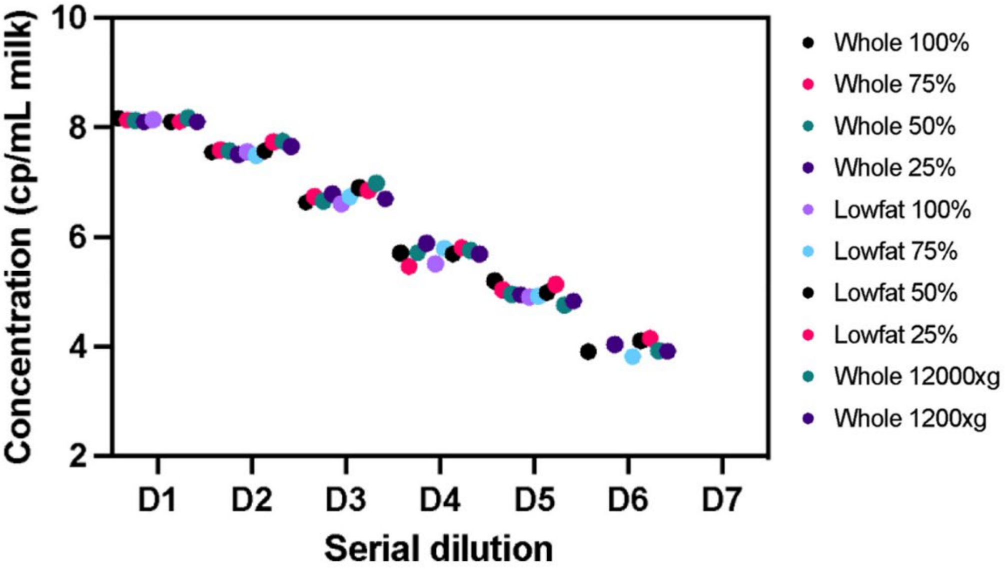

Appendix Figure 4. Evaluation of the MagMAX Prime Viral/Total Pathogen NA extraction kit with serial dilutions of spiked in synthetic H5N1 DNA fragments. Milk was diluted with PBS before spike in and two pre-centrifugation conditions were tested (12000xg for 10 minutes and 1200xg for 30 minutes).

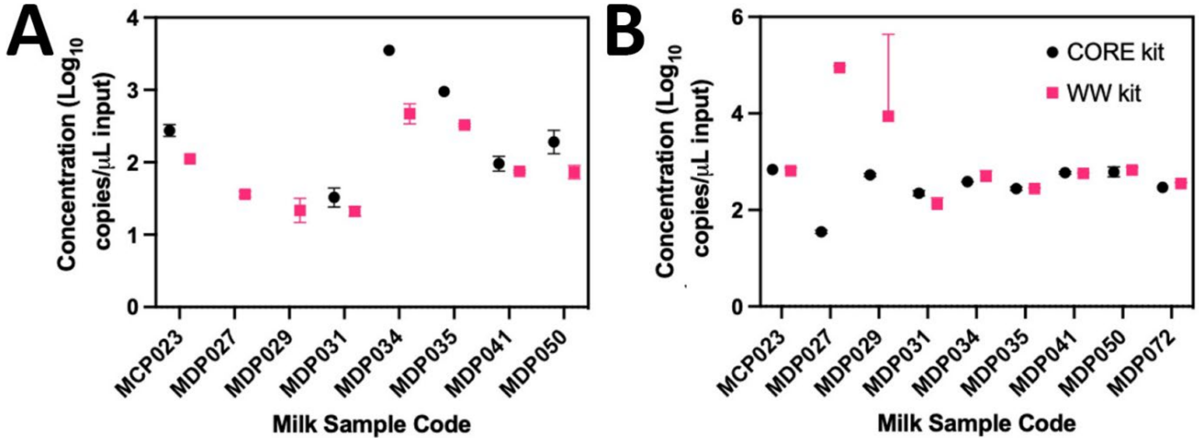

Appendix Figure 5. Comparison of the MagMAX CORE extraction kit versus the MagMAX Wastewater extraction kit on a subset of 8 milk samples for A) H5N1 and B) RnaseP Bovine as measured by dPCR.

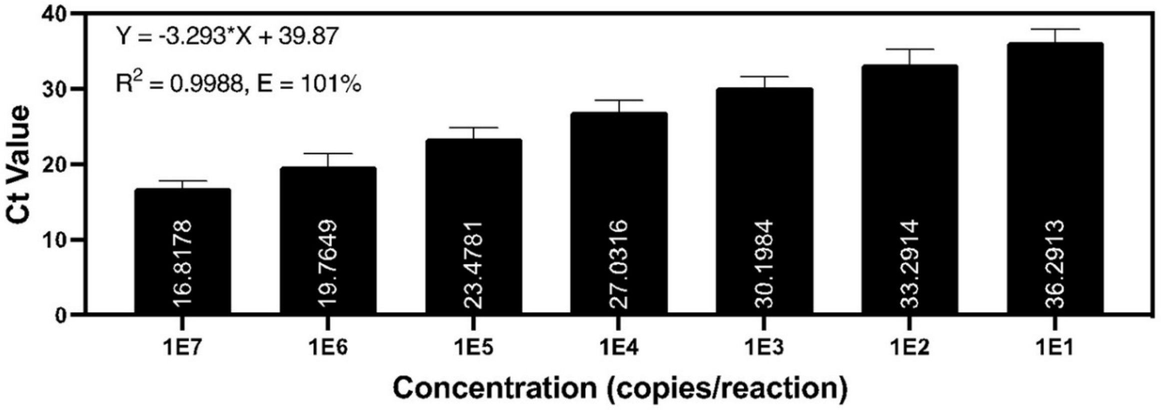

Appendix Figure 6. Characteristics of all qPCR standard curves taken together, showing averages for C_t values of each standard concentration.

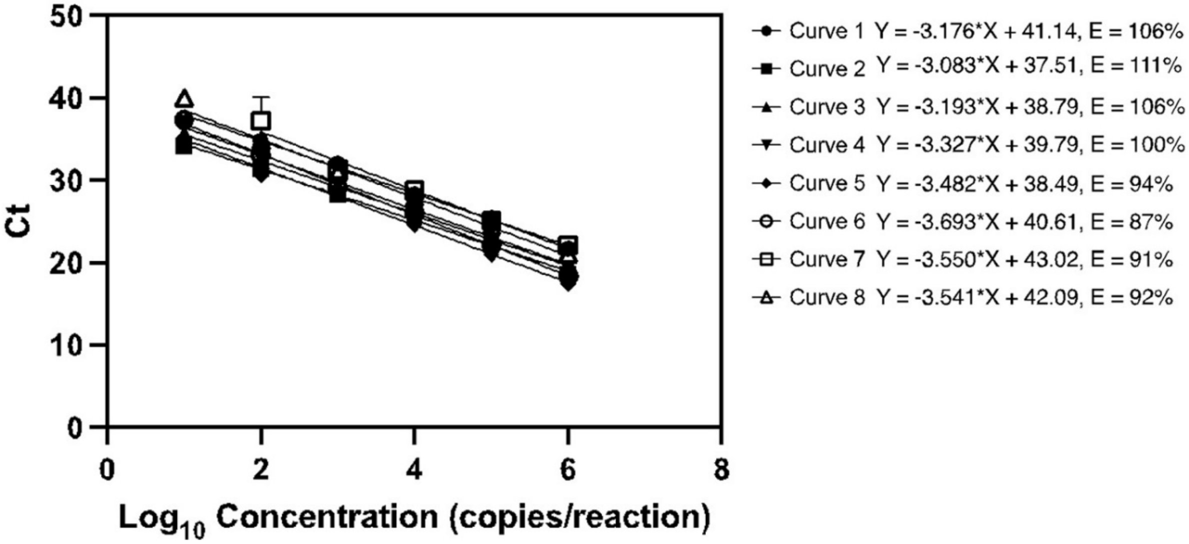

Appendix Figure 7. Standard curves for qPCR throughout the project.

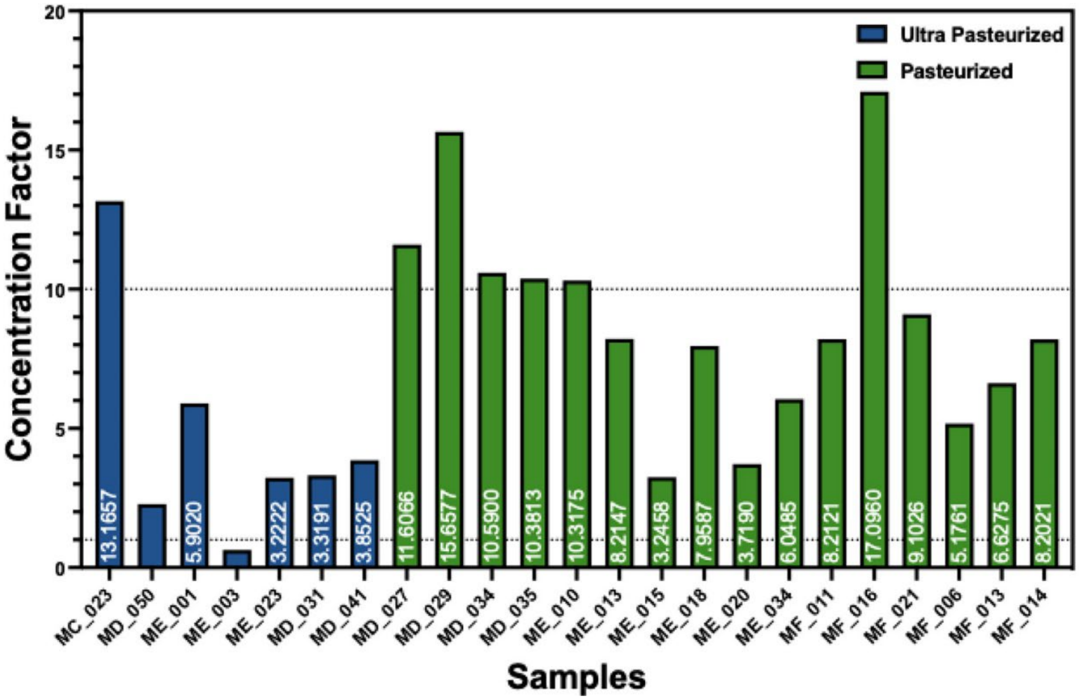

Appendix Figure 8. Concentration factor calculated by concentration in a sample after it was extracted ten times and concentrated divided by the initial concentration of the sample.

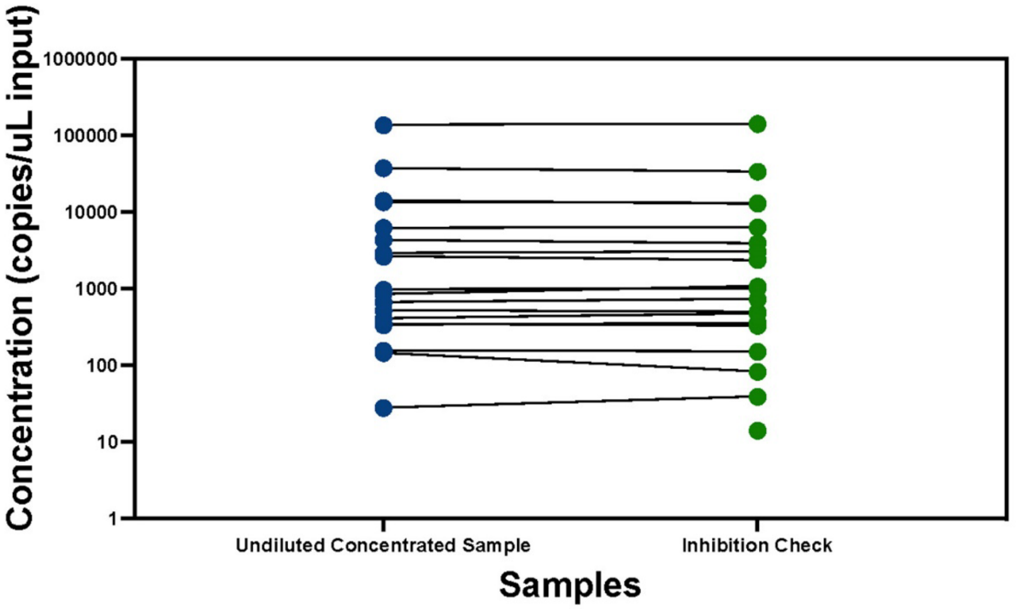

Appendix Figure 9. Inhibition check of concentrated samples as measured by dPCR H5_Taq assay. Inhibition check samples received half the amount of template per reaction. Both reactions were normalized to 1µL of input to be able to compare potential effects of PCR inhibitors.

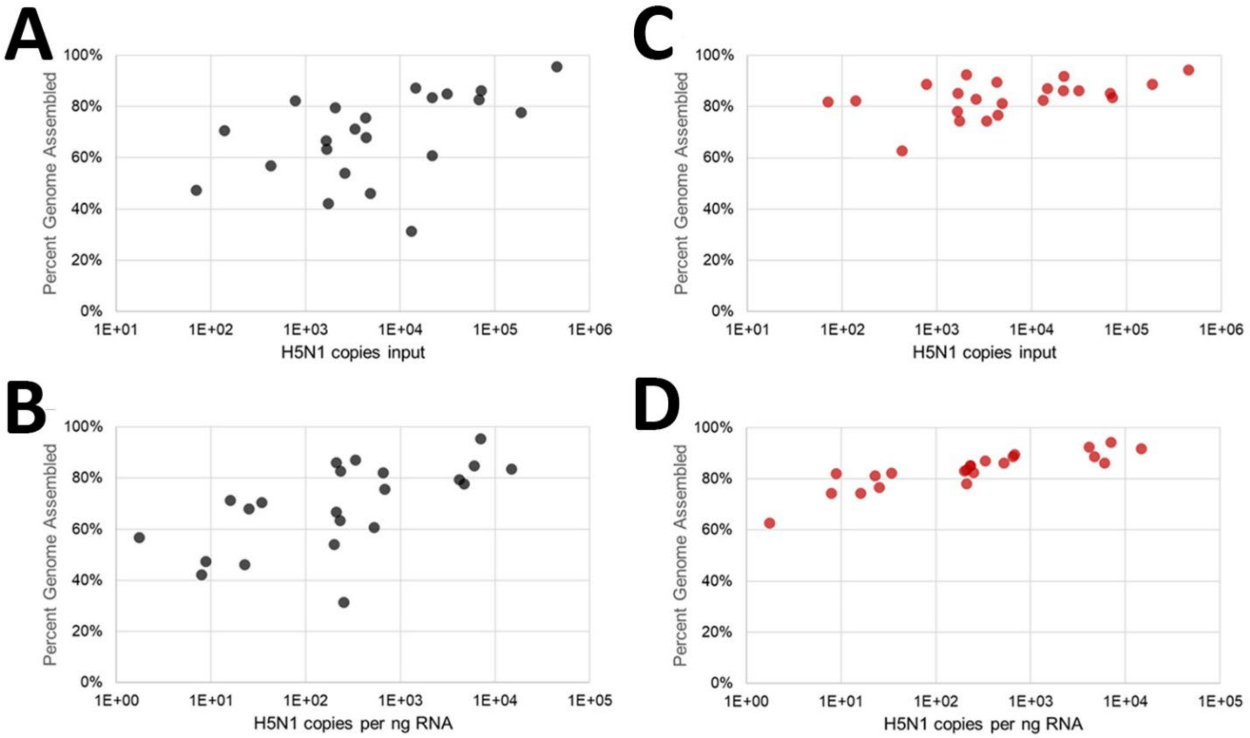

Appendix Figure 10. Percent genomes assembled by Amp-Seq using two cycling conditions to generate H5N1 PCR products performed side-by-side on the same cDNA. A) and C) show the data as a function of H5N1 input copies into library construction whereas B) and D) show the same data as a function of H5N1 copies/ng of RNA. Cycling conditions: A) and B): 30s/98°C; 35x (15s/95°C, 5min/65°C); hold at 4°C; (C) and (D): 1min/98°C; 35x (15s/98°C, 30s/65°C, 45s/72°C); 2min/72°C, hold at 4°C.

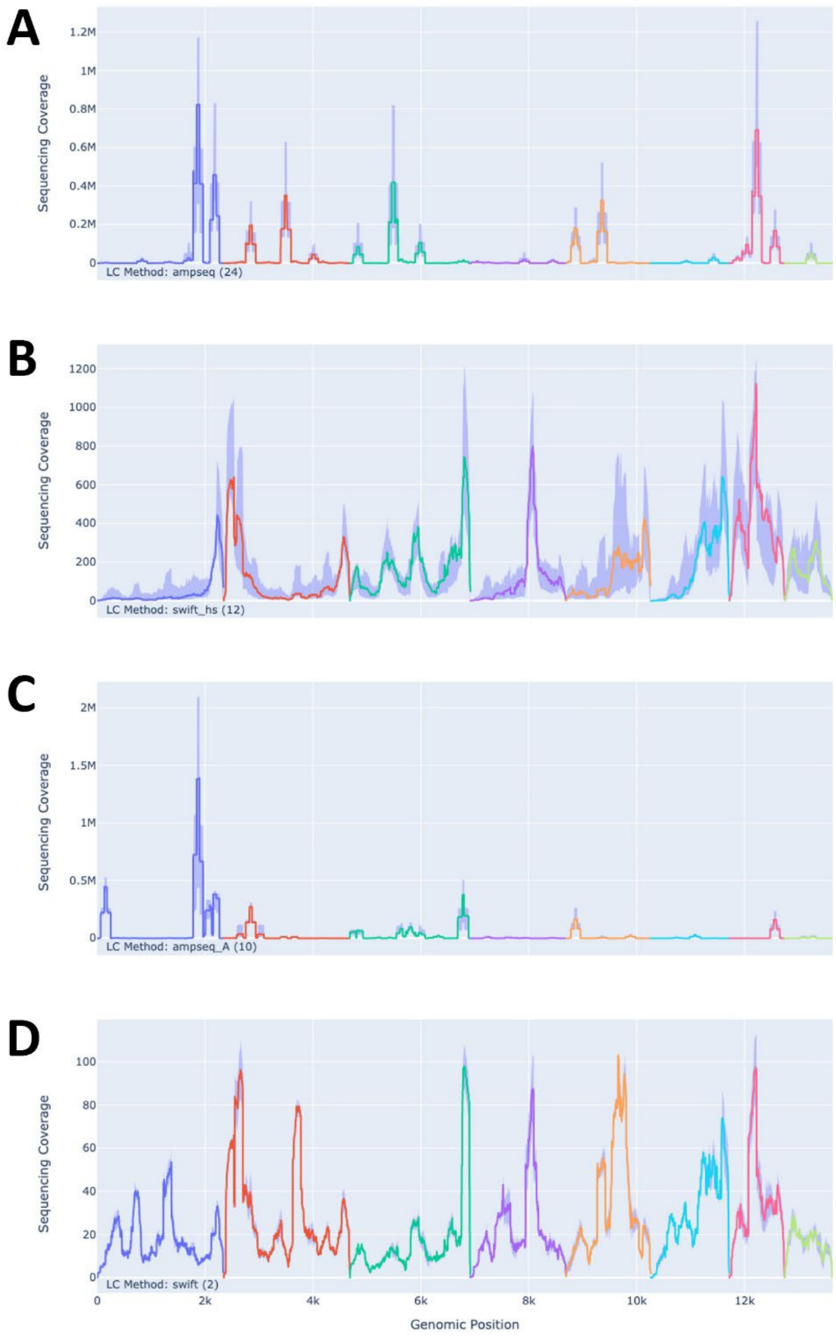

Appendix Figure 11. Sequencing coverage plots, averaged separately for each sequencing method. For each sequencing method, the median sequencing depth across all samples at each genomic position is plotted with a colored line, and 25% - 75% quartile shaded bands are added around the line. Each influenza A genomic segment is plotted in order from segment 1 through 8, each with a different line color. From top to bottom: ampseq refers to the revised Amp-Seq cycling conditions, swift_hs refers to hybrid selected RNA-Seq, ampseq_A refers to the original PCR cycling conditions reported previously, and swift refers to RNA-Seq.

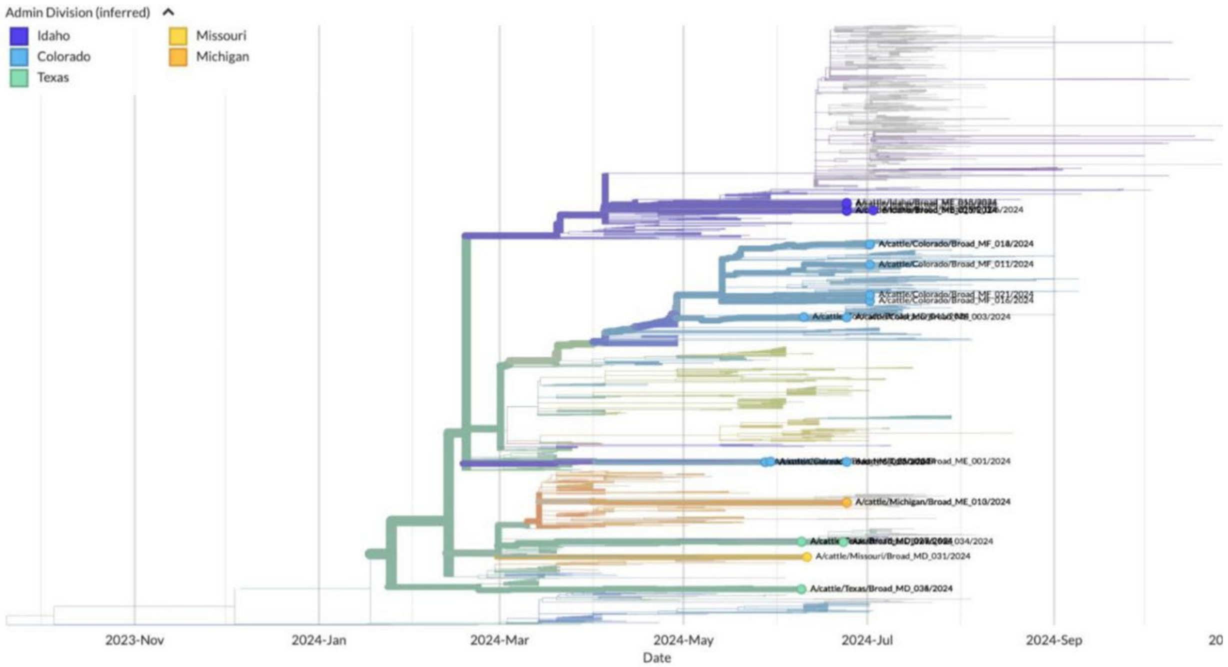

Appendix Figure 12. Phylogenetic tree of all cattle-outbreak-associated H5N1-subtype Influenza A concatenated whole genomes from milk samples, as produced by Louise Moncla and the Nextstrain team. Branches and tips are colored by U.S. state of milk processing plant. Tree is shown on a time-axis to reduce the skew caused by some outlier genomes in the contextual dataset. See Methods for details. Live build can be found at: https://nextstrain.org/avian-flu/h5n1-cattle-outbreak/genome?c = division&d = tree,entropy&f_host = Cattle&f_submitting_lab = Broad%20Institute%20Genomic%20Center%20for%20Infectious%20Diseases,%20Genomic%20Center%20for%20Infectious%20Diseases&m = num_date&p = full.

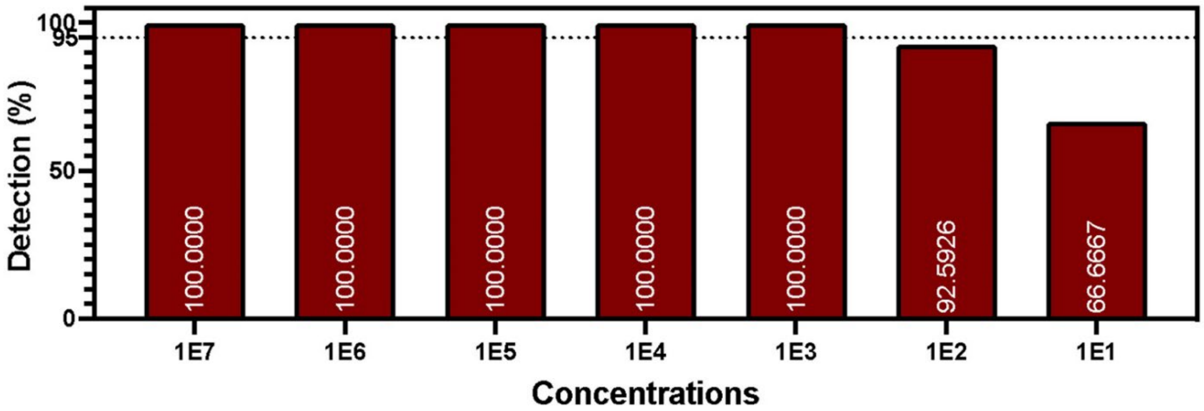

Appendix Figure 13. Detection rate (percentages) of all standard curve dilutions throughout the course of the project.

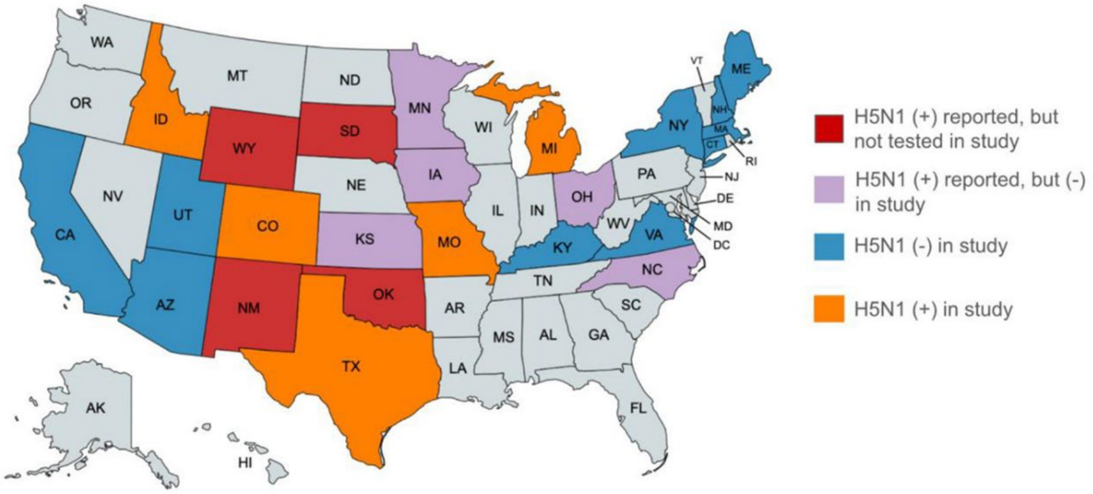

Appendix Figure 14. State map depicting states where milk was sourced from for testing in the current study.
